# Supplementary material for: Sleep and Executive Functioning in Pediatric Traumatic Brain Injury Survivors after Critical Care
Source: Children (Basel). 2022 May 19;9(5):748. doi: 10.3390/children9050748 (PMC9139390; doi:10.3390/children9050748)
Supplement: Supplementary file 1 [file children-09-00748-s001.zip › children-1722811-supplementary.pdf]

Supplementary Table S1. Comparison of analyzed cohort to those with missing or incomplete data for sleep questionnaire or cognitive measures.

	**All Analyzed N = 131 (%)**	**Missing/Incomplete N = 36 (%)**	***p*-value**
Age, Median years (IQR)	11.5 (7.4, 13.8)	13.6 (5.8, 16.6)	0.17
Male sex	78 (60%)	20 (56%)	0.70
Race			0.11
White	94 (72%)	22 (61%)	
Asian	5 (4%)	0 (0%)	
Pacific Islander	3 (2%)	0 (0%)	
African American	1 (1%)	2 (6%)	
American Indian or Alaska Native	1 (1%)	2 (6%)	
More than one race	9 (7%)	4 (11%)	
Unknown or not reported	18 (14%)	6 (17%)	
Hispanic ethnicity	13 (10%)	11 (31%)	0.001
Pre-injury condition, any	38 (29%)	12 (33%)	0.68
Critical Care intervention, any	61 (47%)	24 (67%)	0.04
Inpatient rehabilitation discharge	9 (7%)	7 (19%)	0.02
Glasgow Coma Scale, Median (IQR)	15 (14, 15)	14 (9,15)	0.02
Mechanism of injury			0.01
Fall	36 (28%)	5 (14%)	
Motor vehicle accident	34 (26%)	13 (36%)	
Auto-pedestrian/bike	16 (12%)	4 (11%)	
Bicycle, skateboard, scooter	21 (16%)	4 (11%)	
ATV	9 (7%)	0	
Other blunt	12 (9%)	5 (14%)	
Penetrating	2 (2%)	1 (3%)	
Unknown	1 (1%)	4 (11%)	

Supplementary Table S2. Details of Principle Components Analysis.

Measure	PCA Loading	Inclusion
Combined WISC-V/WAIS-IV Coding	0.74	Retained
Combined WISC-V/WAIS-IV Symbol Search	0.73	Retained
Combined WAIS-IV Digit Span and CMS numbers	0.62	Retained
DKEFS Trails 4 Switching	0.80	Retained
DKEFS Phonemic Verbal Fluency	0.71	Retained
DKEFS Category Fluency	0.80	Retained
CHAMP Lists Immediate	0.77	Retained
CHAMP Lists Delayed	0.74	Retained

Principle components analysis performed on N = 79 patients with complete data in all variables. Combining all participant data yielded a single component solution accounting for 54.7% of the total explained variance. Reasons for missing data in cognitive assessments included complications such as orthopedic injury, vision impairment, and behavioral cooperation precluding completion of some tasks; also, the need for virtual only visits for some patients assessed during the COVID-19 pandemic.

Supplementary Table S3. Average cognitive outcomes and brain injury severity assigned by the Glasgow Coma Scale.

	**All Mean (SD)**	**Mild Mean (SD)**	**Mild Complicated Mean (SD)**	**Moderate Mean (SD)**	**Severe Mean (SD)**	***p*-value (ANOVA)**
BRIEF-GEC, N = 100	53.89 (12.43)	53.27 (11.85)	53.31 (13.39)	53.50 (14.28)	59.78 (10.15)	0.53
Neurocognitive Index, N = 79	-0.46 (1.01)	0.004 (0.99)	-0.004 (1.05)	-0.32 (1.0)	-0.16 (1.10)	0.87
Numbers combined, N = 108	8.40 (2.73)	8.81 (2.53)	7.87 (2.59)	8.30 (2.98)	8.50 (3.68)	0.47
Lists Immediate, N = 110	8.50 (2.70)	8.33 (2.41)	8.98 (2.60)	6.67 (2.61)	9.42 (3.45)	0.04
Lists Delayed, N = 111	8.60 (3.29)	8.67 (3.07)	9.15 (3.07)	7.67 (3.50)	7.50 (4.44)	0.33
DKEFS number letter switching, N = 85	7.21 (4.05)	7.37 (4.37)	7.85 (3.74)	6.13 (3.87)	6.00 (3.84)	0.50
DKEFS category fluency, N = 88	9.69 (3.53)	10.08 (3.31)	10.14 (3.44)	8.56 (4.19)	8.38 (3.80)	0.31
DKEFS letter fluency, N = 87	8.14 (2.81)	8.29 (2.75)	8.85 (2.85)	6.33 (2.69)	7.46 (2.60)	0.10
Combined coding, N = 105	8.31 (3.09)	8.62 (3.31)	8.36 (3.10)	8.33 (3.20)	7.00 (1.91)	0.46
Combined symbol search, N = 105	9.59 (3.39)	9.76 (3.26)	9.78 (4.03)	9.08 (2.11)	8.92 (2.97)	0.82
Word reading, N = 111	97.97 (16.02)	97.30 (13.56)	100.44 (17.90)	91.50 (9.83)	98.67 (22.14)	0.39
SDSC Domain T-score, N = 131						
Total score	56.92 (13.67)	57.61 (14.83)	57.92 (12.53)	53.25 (16.76)	53.86 (9.57)	0.58
Initiation and maintenance	62.97 (16.12)	63.61 (15.95)	64.16 (16.37)	58.00 (14.45)	60.50 (14.45)	0.61
Breathing	50.47 (9.98)	50.57 (10.33)	49.80 (8.61)	50.83 (6.95)	52.07 (15.01)	0.90
Arousal	54.48 (14.53)	55.52 (14.99)	56.59 (16.78)	48.83 (4.28)	47.79 (2.94)	0.11
Sleep wake transition	54.89 (14.76)	55.20 (15.36)	56.33 (15.25)	52.42 (15.11)	50.71 (9.73)	0.59
Excessive somnolence	49.67 (8.42)	49.91 (9.64)	49.78 (7.34)	48.83 (9.66)	49.07 (6.06)	0.97
Hyperhidrosis	49.50 (8.56)	50.38 (10.06)	49.00 (7.42)	48.08 (7.57)	48.93 (7.00)	0.77

Supplemental Table S4. Correlation between cognitive outcomes, injury severity measures, and sleep subscale T-scores

	GEC	NCI	Numbers combined	Lists immediate	Lists delay	DKEFS number letter switch	DKEFS category fluency	DKEFS letter fluency	Coding	Symbol search	Word reading	GCS	ISS	AIS head
DIMS	.48**	-.28*	-.06	-.11	-.10	-.20	-.18	.08	-.23*	-.02	-.10	.11	.11	-.04
SBD	.20*	-.20	-.06	-.15	-.10	-.17	-.19	-.15	-.06	-.06	-.11	-.03	-.03	.10
DA	.27*	-.06	.05	.06	.10	.04	-.05	-.01	.12	.20*	.05	.19*	.19	-.002
SWTD	.33**	-.16	-.10	-.05	-.06	-.10	-.09	.10	-.02	-.04	-.001	.04	.04	-.03
DES	.47**	-.31**	-.24*	-.19*	-.24*	-.14	-.16	-.06	-.14	-.08	-.17	-.05	-.05	.01
SH	.27**	-.34**	-.13	-.19*	-.16	-.28*	-.21*	-.16	-.28**	-.12	-.22*	.12	.12	-.09
SDSC Total	.52**	-.33**	-.12	-.10	-.13	-.21	-.20	.04	-.18	-.05	-.09	.07	.07	.02
GCS	-.16	.10	.03	.03	.19	.20	.23*	.16	.17	.14	.11	--	-.37**	-.33**
ISS	.20*	-.18	-.15	-.09	-.13	-.24*	-.31**	-.11	-.11	-.11	-.02	-.37**	--	.75**
AIS head	.13	.04	.03	.14	.10	.002	-.08	.02	.11	-.004	.14	-.33**	.75**	--

Spearman correlation coefficients. *Significant at $p < 0.05$, **Significant at $p < 0.01$.
Sleep Disturbances Scale for Children (SDSC) Domain T-scores used. DIMS: Disorders of Initiation and Maintenance domain; SBD: Sleep breathing disorders domain; DA: Disorders of arousal domain; SWTD: Sleep wake transition disorder domain; DES: Disorders of excessive somnolence domain; SH: Sleep hyperhidrosis domain; SDSC Total: Sleep Disturbances Scale for Children total score
GEC: Global Executive Composite; NCI: Neurocognitive Index; DKEFS: Delis Kaplan Executive Function System
GCS: Glasgow Coma Scale; ISS: Injury Severity Score; AIS: Abbreviated Injury Scale.
